# Improving wheat to remove coeliac epitopes but retain functionality

**DOI:** 10.1016/j.jcs.2015.06.005

**Published:** 2016-01

**Authors:** Peter R. Shewry, Arthur S. Tatham

**Affiliations:** a Rothamsted Research, Harpenden, Hertfordshire AL5 2JQ, UK; b University of Reading, Whiteknights, Reading, Berkshire RG6 6AH, UK; c Cardiff School of Heath Sciences, Cardiff Metropolitan University, Llandaff Campus, Western Avenue, Cardiff CF5 2YB, UK

**Keywords:** Wheat, Coeliac disease, Coeliac-safe, Breeding

## Abstract

Coeliac disease is an intolerance triggered by the ingestion of wheat gluten proteins. It is of increasing concern to consumers and health professionals as its incidence appears to be increasing. The amino acid sequences in gluten proteins that are responsible for triggering responses in sensitive individuals have been identified showing that they vary in distribution among and between different groups of gluten proteins. Conventional breeding may therefore be used to select for gluten protein fractions with lower contents of coeliac epitopes. Molecular breeding approaches can also be used to specifically down-regulate coeliac-toxic proteins or mutate coeliac epitopes within individual proteins. A combination of these approaches may therefore be used to develop a “coeliac-safe” wheat. However, this remains a formidable challenge due to the complex multigenic control of gluten protein composition. Furthermore, any modified wheats must retain acceptable properties for making bread and other processed foods. Not surprisingly, such coeliac-safe wheats have not yet been developed despite over a decade of research.

## Highlights

• Coeliac disease is of increasing concern as its incidence appears to be increasing.
• Over 30 amino acid sequences (coeliac epitopes) have been defined.
• Coeliac epitopes differ in their distribution between wheat gluten proteins.
• Transgenesis can be used to reduce coeliac-toxic proteins and coeliac epitopes.
• This can be exploited to develop “coeliac-safe” wheats.

## 1 Introduction

### 1.1 The importance of wheat in human nutrition and health

Cereals are the most widely grown and consumed staple foods in the world, with three species alone (maize, rice and wheat) accounting for about 90% of the total production. Although wheat is third in order of total production, with about 713 m tonnes grown in 2013 compared with 745 m tonnes of rice and 1017 m tonnes of maize (http://faostat.fao.org/site/339/default.aspx), it has the widest geographical distribution, being grown and consumed as a staple food between 67°N in Scandinavia and 45°S Argentina (Feldman, 1995), and in both highly industrialised western economies (Western Europe, North America) and in developing economies (China, Brazil, India). Hence it can be argued that wheat is the most important crop in the world in its global impact on human nutrition. Wheat consumption is also increasing globally, For example, the availability of wheat as a %age of total kCal in food increased from 11.85 in 1961 to 24.41% in 2011 in India, and from 12.20% to 17.83% in China (FAO Food Balance Sheets http://faostat3.fao.org/faostat-gateway/go/to/download/FB/FBS/E). Furthermore, demand is increasing dramatically in industrialising countries in which the production of wheat is limited by climatic conditions, such as West Africa (e.g. an increase from 0.89% to 6.64% of the total available kCal in Nigeria between 1961 and 2011).

Within these countries wheat makes important contributions to diet and health, particularly the provision of dietary fibre, B vitamins (notably vitamins B1, B2, B3, B6 and B9 (folates)) and mineral micronutrients (notably Fe, Zn, Se). This contribution is most readily demonstrated for developed economies where accurate data are available on food intakes. For example, the UK National Diet and Nutrition Survey (NDNS) showed that cereals account for 31% (and breads for 10–12%) of the total daily intake of energy in adults between the ages of 19 and 64, 23% (breads for 10–11%) of the total daily intake of protein, 37–40% (breads for 18–21%) of the total daily intake of non-starch polysaccharides (ie dietary fibre), 38–40% (breads for 15–16%) of the total daily intake of Fe and 27% (breads 12%) of the total daily intake of folates (https://www.gov.uk/government/publications/national-diet-and-nutrition-survey-results-from-years-1-to-4-combined-of-the-rolling-programme-for-2008-and-2009-to-2011-and-2012).

Vitamins and minerals have long been known to be essential for human health, while cereal dietary fibre has been shown to reduce the risk of a range of chronic diseases (including cardio-vascular disease, type 2 diabetes and colo-rectal cancer (Topping, 2007, Buttriss and Stokes, 2008, Anderson et al., 2009a, Anderson et al., 2009b, Aune et al., 2011, Threapleton et al., 2013, Lafiandra et al., 2014, SACN, 2014). In addition, wheat is rich in a range of phytochemicals, notably phenolic acids and other phenolic compounds, which have been reported (but not conclusively established) to have benefits in reducing the risk of chronic diseases. Hence, restricting the intake of wheat in the diet can have serious consequences for the intake of essential nutrients and other beneficial components unless equivalent sources of these are provided.

Almost all of the wheat consumed by humans undergoes extensive processing before consumption. This usually comprises two stages. Firstly, the grain is milled to give fine particles and, in most cases, to separate the starch-rich endosperm (which is the origin of white flour) from the outer layers (aleurone, pericarp and testa) and germ (which together form the bran). Secondly, the flour is processed into various foods, most commonly bread but also other baked goods (cakes, biscuits), noodles (bread wheat) and pasta (durum wheat) and breakfast cereals. Wheat flour, and gluten (see below), are also widely used as ingredients in the food industry.

### 1.2 Wheat gluten proteins

The use of wheat for most food products in underpinned by the gluten proteins. These correspond to the major group of grain storage proteins which are deposited in the starchy endosperm cells to support germination and seedling growth. They account for up to 80% of the total grain proteins, which in turn account for between 10 and 15% of the dry weight of grain grown commercially. These proteins form a continuous matrix surrounding the starch granules in the mature starchy endosperm cells, and are brought together to form a continuous network when flour is mixed with water to give dough. This network confers a unique combination of elasticity and viscosity which enable the dough to be processed into the range of products discussed above. Although related proteins are present in other temperate cereals (barley, rye and oats) they do not share the same properties and it is necessary to blend them with wheat flour to make products which are acceptable to most consumers. It is therefore crucial that any modifications that are made to the amount and composition of the gluten proteins should also be considered in relation to their effects on the biophysical and functional properties of dough.

Wheat gluten proteins are traditionally classified into two groups based on their solubility. The gliadins are readily extracted from flour with alcohol:water mixtures, such as 60% (v/v) ethanol or 50% (v/v) propan-1-ol, while the glutenins were traditionally extracted with dilute acid or alkali. However, these fractions contain related proteins and the differences in solubility are determined by their presence as monomers or polymers. Thus, the gliadin fraction comprises mainly proteins which are present as monomers, with small amounts of polymeric components, while the glutenins comprise “subunits” assembled into high molecular mass polymers stabilized principally by inter-chain disulphide bonds. When these disulphide bonds are reduced the monomeric glutenin subunits resemble the gliadins in being soluble in alcohol:water mixtures. Hence, the protein subunits present in both fractions correspond to alcohol-soluble prolamin proteins as defined in the classic studies of Osborne (1924).

Gluten protein fractions comprise many individual components, with a high level of allelic variation in composition between cultivars. The individual components can be classified on the basis of their amino acid compositions and sequences into three families, which have been called the sulphur-rich (S-rich), sulphur-poor (S-poor) and high molecular weight (HMW) prolamins (Shewry et al., 1986).

**Fig. 1:**
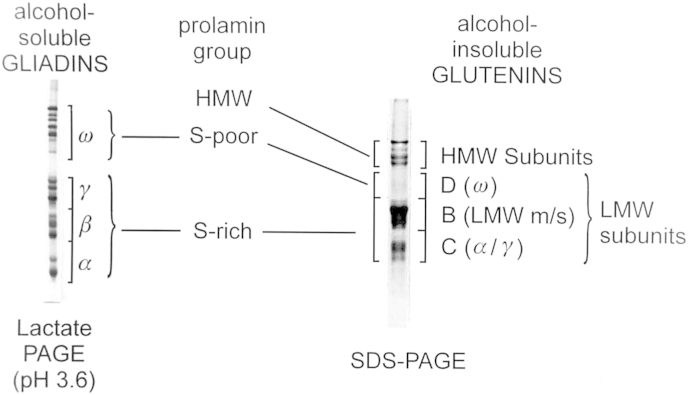
The groups of gliadin and glutenin proteins separated by electrophoresis at low pH and SDS-PAGE, respectively. Taken from Shewry et al. (1999) with permission.

The gliadins are traditionally divided based on their mobility in electrophoresis at low pH into three groups: the S-rich α-type gliadins and γ-type gliadins (which contain three and four inter-chain disulphide bonds, respectively) and the S-poor ω-gliadins (which lack cysteine residues and hence do not form disulphide bonds) (Fig. 1). Similarly, the glutenin subunits are separated by sodium dodecylsulphate polyacrylamide electrophoresis (SDS-PAGE) into low molecular weight subunits (LMW subunits) and high molecular weight subunits (HMW prolamins) of glutenin (Fig. 1). The LMW and HMW subunits form inter-chain disulphide bonds which stabilise the glutenin polymers, while intra-chain disulphide bonds are also formed by LMW subunits and at least some HMW subunits.

Whereas most of the LMW subunits are S-rich and form a distinct group within the S-rich family (B-type LMW subunits), the fraction also contains small proportions of components that are closely related in sequence to the α-, γ- and ω-gliadins. These components correspond to “mutant” forms of gliadins in which the presence of one or two additional cysteine residues allows their incorporation into polymers. The LMW subunits related to ω-gliadins correspond to one or more bands of slightly higher molecular weight than the B-type components (called D-type LMW subunits) and those related to γ- and ω-gliadins correspond mainly to a group of bands of lower molecular weight (C-type LMW subunits). The B-type LMW subunits can be further sub-divided based on their N-terminal amino acids: M (methionine) or S (serine). There are also clear differences between the amino acid sequences of the ω-gliadins encoded by chromosomes 1A and 1D and those encoded by chromosome 1B (also called ω5-gliadins): these relate to their toxicity in coeliac disease and are discussed below.

The HMW subunits are further divided into x-type and y-type based on their mobility on SDS-PAGE. These two types also differ in their contents and distributions of cysteine residues.

**Fig. 2:**
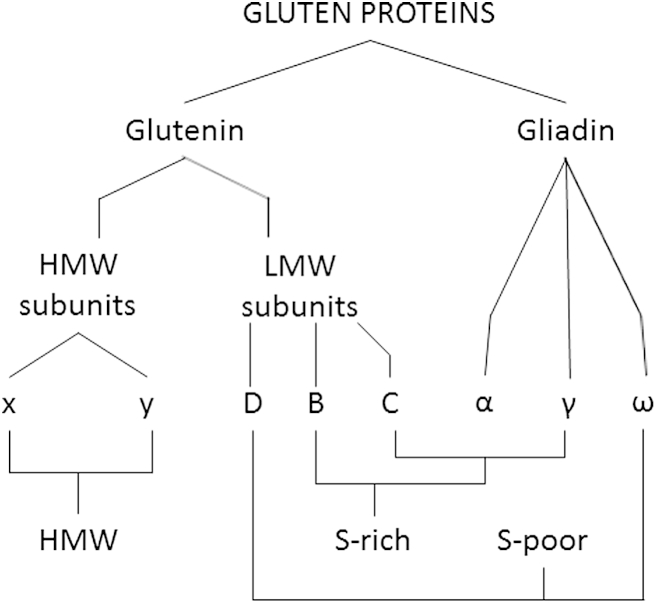
Schematic summary of the classification of gluten proteins. See Fig. 1 and the text for details.

This brief summary is based on a considerable volume of research and the reader is referred to Shewry et al., 1999, Shewry et al., 2003a, Shewry et al., 2003b, Shewry et al., 2009 and Payne (1987) for more detailed discussions and references. This rather complex classification is summarized for clarity in Fig. 2.

### 1.3 Wheat gluten protein genes and expressed proteins

Genetic and molecular analyses indicate that the individual gluten proteins are encoded by multiple genes at complex loci. The classical genetics has been reviewed in detail by Shewry et al. (2003a). The HMW subunits of glutenin are encoded by three loci on the long arms of the group 1 chromosomes (*Glu-A1*, *Glu-B1, Glu-D1*), each comprising two genes encoding one x-type and one y-type HMW subunit. Similarly, the α-type gliadins are encoded by three loci on the sort arms of the group 6 chromosomes (*Gli-A2*, *Gli-B2*, *Gli-D2*), 6B, 6D), but these loci are more complex and may together comprise over 50 genes (most of which do not appear to be expressed) (Anderson and Greene, 1997, Van Herpen et al., 2006). The genetic control of the λ-type gliadins, ω-gliadins and LMW subunits is more complex, with three major loci for each (Gli-A1, *Gli-B1*, *Gli-D1* and *Glu-A3*, *Glu-B3*, *Glu-D3*, respectively) located on the short arms of the group 1 chromosomes. However, a number of minor loci encoding both gliadins and LMW subunits have been mapped to the same chromosome arms.

The major gliadin and LMW subunit loci are clearly multigenic. Qi et al. (2009) reported 29 functional λ-gliadin genes in Chinese Spring wheat but Anderson et al. (2013) only 11 functional genes in the same variety. Multiple ω-gliadin genes are also present at the *Gli-3* loci (Sabelli and Shewry, 1991, Anderson et al., 2009a, Anderson et al., 2009b). Cassidy et al. (1998) reported 17 genes for B-type LMW subunits; the genes encoding C-type and D-type subunits can be expected to be included in those reported for the corresponding gliadin gene families, as discussed by Anderson et al., 2009a, Anderson et al., 2009b for the D-type LMW subunits and ω-gliadins.

More realistic estimates of the numbers of expressed gluten proteins are provided by proteomic studies. For example, Dupont et al. (2011) reported 5 HMW subunit proteins, 22 LMW subunit proteins, 13 γ-gliadins, 13 α-gliadins and 7 ω-gliadins in the cultivar Butte 86.

## 2 Coeliac disease and the identification of toxic motifs in wheat

### 2.1 Intolerance and allergy to wheat

Coeliac disease (CD) is one of a number of diseases associated with the ingestion of the gluten proteins of wheat and related prolamins of barley, rye and, in some individuals, oats. CD is classified as a T cell-mediated autoimmune disease (Sapone et al., 2012) in which the ingestion of prolamins results in the flattening of the villi in the intestinal tract and the subsequent malabsorption of nutrients. It is one of the commonest food intolerances, with an incidence of approx. 1% of the population (Biagi et al., 2010). Two much rarer forms of intolerance which are related to CD have been described: these are dermatitis herpetiformis (DH) and gluten ataxia (GA). DH is described as the skin manifestation of CD, presenting as a rash and IgA deposits in the skin and affects one to six per 10,000 of the population (Salmi et al., 2011). In GA, antibodies produced against the prolamins cause damage to the cerebellum (the part of the brain responsible for balance and motor control) (Hadjivassilou et al., 2008). For all three conditions the only treatment is a strict gluten free diet (Sapone et al., 2012).

Cereal allergies also involve the immune system, but in this case the response is mediated by the production of IgE. Cereal allergies comprise respiratory allergy (bakers' asthma), food allergy, contact urticarial and wheat-dependent exercise-induced anaphylaxis (WDEIA). In addition to prolamins, reported allergens include albumins, globulins and other proteins of wheat (Tatham and Shewry, 2008). Of the cereal allergies, only WDEIA has been characterised in detail, showing the presence of epitopes in the proline and glutamine-rich repetitive domains of the ω5-gliadins (B genome encoded) and HMW subunits of wheat glutenin (Matsuo et al., 2004). In other forms of cereal allergy, the causative allergens are more heterogeneous, comprising prolamin and non-prolamin proteins. These conditions will not be considered further here.

CD is the commonest food intolerance and appears to be increasing in prevalence, although the reasons for this are currently unclear (Catassi et al., 2007). However, the increasing consumption of wheat in the place of traditional foods (non-gluten containing cereals such as maize and sorghum, and tubers) in Africa and Asia (discussed earlier) may lead to an increase in CD in these countries. CD is strongly associated with specific genetic backgrounds, particularly with the genes encoding the HLA-DQ2 and DQ8 serotype groups, with 95% of CD patients exhibiting the DQ2 serotype class (see Scherf, Koehler & Wieser, this volume).

### 2.2 Identification of coeliac disease epitopes

Currently thirty-one, nine amino acid peptide sequences in the prolamins of wheat and related species have been defined as being coeliac toxic: these are often referred to as coeliac “epitopes”. However, mapping is incomplete and the number of distinct epitopes a matter of on-going discussion (Sollid et al., 2012). These epitopes are located in the repetitive domains of the prolamins, which are proline and glutamine-rich, and the high levels of proline in their sequences may reduce their susceptibility to protease activity in the GI tract. The prolamin-reactive T cells (T lymphocytes) of CD patients also recognise these epitopes to a greater extent when specific glutamine residues in their sequences have been deamidated to glutamic acid by a tissue transglutaminase (tTG2). This binding enables the formation of a stable peptide-MHC complex, which is important in the anti-prolamin T-cell response (Sollid et al., 2012).

The majority of coeliac toxic peptides have been identified from *in vitro* studies using peptides cultured with T cell lines or T cell clones derived from the biopsied small intestinal mucosa of CD patients (Carmarca et al., 2012). Anderson et al. (2000) developed an *in vivo* method, reporting that T cells recognised the same prolamin epitopes in the peripheral blood of CD patients after oral gluten challenge as those identified by organ culture. The current list of T cell prolamin epitopes recognised by T cells is a combination of peptides identified by these two methods (Sollid et al., 2012). Camarca et al. (2009) reported considerable variation in the T cell responses of fourteen coeliac patients, which would indicate that there are probably far more active epitopes than listed by Sollid et al. (2012).

Tye-Din et al. (2010) reported a hierarchy of coeliac-stimulating peptides after challenge with wheat, barley and rye. This showed that the immunodominant sequence after wheat challenge corresponds to a well-characterised 33 residue peptide from α-gliadin that contains the overlapping T-cell epitopes DQ2.5-glia-α1a, b and DQ2.5-glia-α2. This “33-mer” is resistant to gastrointestinal digestion (with pepsin and trypsin) and was initially identified as the major coeliac toxic peptide in the gliadins. Whereas most previous studies concentrated on wheat, Tye-Din et al. (2010) extended studies to barley and rye and reported that the peptides that stimulated T cells were the same among patients that consumed the same cereal, but were different after wheat, barley and rye ingestion. However, analysis after oral challenge with a mixture of prolamin fractions from wheat, barley and rye showed that sequences derived from S-poor prolamins (ω-gliadins, ω-secalins and C hordeins) were immunodominant. Coeliac toxic peptide epitopes from barley and rye are located in the S-poor C hordeins and ω-secalins, respectively (Sollid et al., 2012). Hence, Tye-Din et al. (2010) concluded that the S-poor derived epitopes DQ2.5-gli-ω1 and DQ2.5-gli-ω2 were also immunodominant in HLA-DQ2 associated CD. Furthermore they reported that T cells specific for a restricted number of peptides accounted for most prolamin specific T cell responses.

### 2.3 Distribution of coeliac disease epitopes

**Fig. 3:**
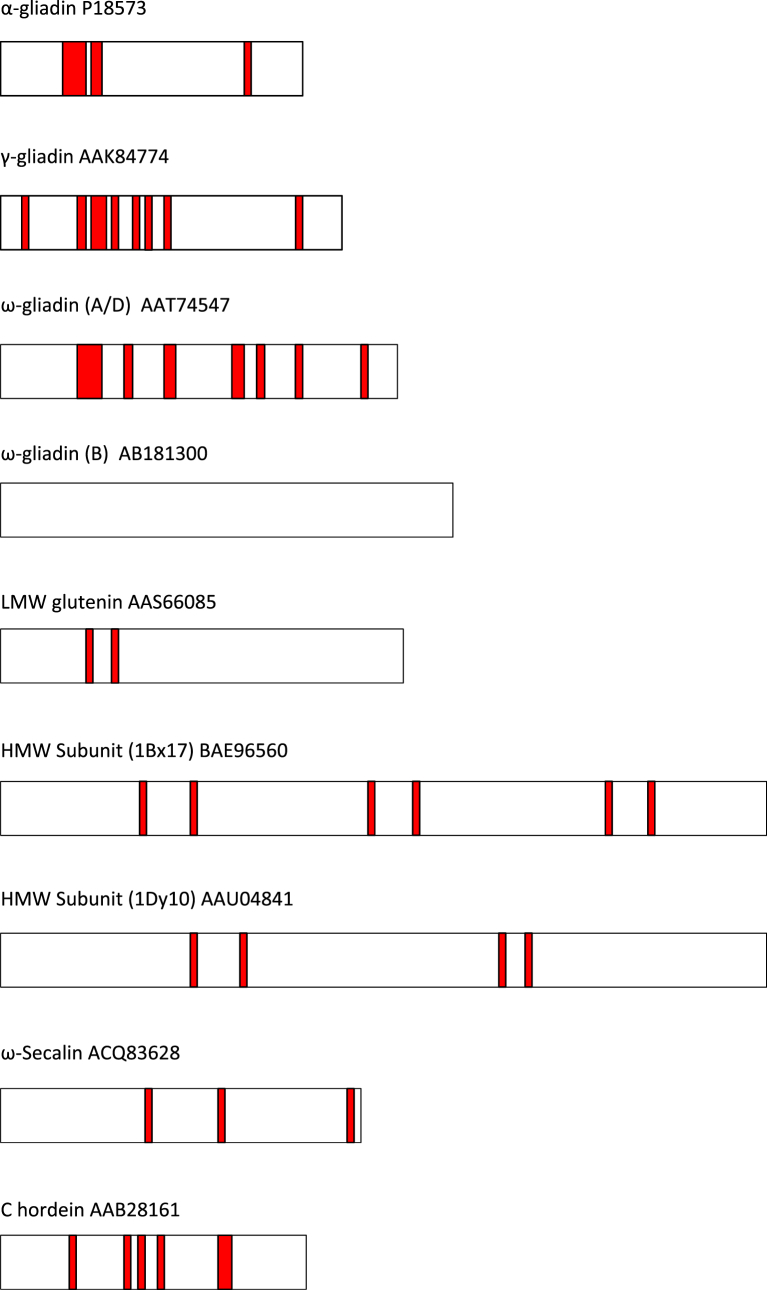
Coeliac toxic epitope distribution in representative prolamins identified by GenBank accession code and T-cell epitopes from Sollid et al. (2012). **α-gliadin** P18573: DQ2.5-glia-α1a, DQ2.5-glia-α1b, DQ2.5-glia-α2 & DQ8-glia-α1. **γ-gliadin** AAK84774: DQ2.5-glia-ω1/hor-1/sec-1, DQ8-glia-γ1a, DQ8-glia-γ2, DQ8-glia-γ4c & DQ8-glia-γ5. **ω-gliadin (A/D)** AAT74547: DQ2.5-glia-γ5, DQ8-glia-γ1a, DQ2.5-glia-ω1/hor-1/sec-1, DQ8-glia-γ1b & DQ2.5-glia-γ3. **ω-gliadin (B)** AB181300 no coeliac toxic epitopes present. **LMW subunit** AAS66085:DQ2.5-glut-L1. **HMW Subunit (1Bx17)** BAE96560: DQ8.5-glut-H1. **HMW Subunit (1Dy10)** AAU04841: DQ8.5-glut-H1. **ω-secalin** ACQ83628: DQ2.5-glia-γ5 & DQ2.5-glia-ω1/hor-1/sec-1**. C hordein**: DQ2.5-glia-γ5, DQ8-glia-γ1a & DQ2.5-glia-ω1/hor-1/sec-1.

Information on the frequency, distribution and immunodominance of coeliac toxic epitopes is required to underpin the use of conventional breeding or genetic engineering to develop lines with reduced CD toxicity. Table 2 shows the current list of T-cell epitopes for wheat, barley and rye (Sollid et al., 2012) and Fig. 3 their distribution in the amino acid sequences of representative prolamins. The sequences were selected from the GenBank database and mapped for the un-deamidated forms of the coeliac toxic peptide identified by Sollid et al. (2012). Although these sequences were selected as representative of the different types of wheat prolamin they do not feature all of the epitopes listed by Sollid et al. (2012).

In the α-type gliadins the epitopes are predominantly distributed in the N-terminal repetitive domain while in the γ-type gliadins they are more widely distributed with a higher frequency of occurrence. The repetitive domain of the γ-type gliadin sequence shown in Fig. 3 contains the epitope (DQ2.5-glia-ω1) associated with the S-poor prolamins and epitopes for both HLA-DQ2 and DQ8 serotype groups.

There is a marked difference in the distributions of epitopes between the ω-gliadins encoded by the A/D genomes and B genome of bread wheat: whereas epitopes are distributed throughout the sequences of the chromosome A/D encoded ω-gliadins they are absent from the B-encoded ω-gliadins (ω5-gliadins). However, Vader et al. (2002) reported a T-cell stimulatory sequence (Glu-5: QIPQQPQQF) that is ω5-gliadin-derived and present at multiple sites. It is therefore unlikely that this class of ω-gliadins is devoid of coeliac toxic peptides. The presence of shared epitopes in the chromosome A/D encoded ω-gliadins and the γ-gliadins is to be expected given the similarity between the repetitive domains of these two types of protein. Similarly, the ω-secalins of rye and C hordeins of barley are closely related to the A/D genome encoded ω-gliadins of wheat and show similar distributions of epitopes (the DQ2.5-gli-ω1, DQ2.5-hor-1 and DQ2.5-sec-1 epitopes being homologous). This similarity has been proposed as the basis for the cross-reactivity between wheat, barley and rye prolamins, as barley and rye do not contain close homologues of the α-gliadins of wheat and α-gliadin type epitopes are therefore absent (Tye-Din et al., 2010). The LMW subunit sequence AAS66085 contains one epitope (DQ2.2-glut-L1) as do the x- and y-type HMW subunits of glutenin (DQ8.5-glut-H1), with both epitopes being repeated in the sequences. The number of DQ8 epitopes is less than those of DQ2 epitopes in all sequences apart from the HMW subunits, where they are the only epitopes present and are distributed throughout the sequences.

Mutagenesis has been used to determine the importance of individual amino acid residues in coeliac epitopes, and identify substitutions that eliminate activity. For example, Anderson et al. (2006) mutated the sequences DQ2.5-glia-α1a and DQ2.5-glia-α2 and found that single substitutions abolished activity. These studies are important as the provide a basis for identifying naturally occurring “coeliac-safe” variants of prolamins in wheat lines, and targets for molecular breeding approaches. These are discussed below.

## 3 Reducing coeliac toxicity by exploiting natural variation in cultivated wheat and wild relatives

### 3.1 Genetic diversity of wheat

Wheat occurs in a range of diploid, tetraploid and hexaploid forms (summarised in Table 1). The earliest cultivated forms were the A genome diploid einkorn (*T. monococcum* var *monococcum*) and tetraploid emmer (*T. turgidum* var. *dicoccum*) with the A and B genomes. These are closely related to wild forms: diploid *T. monococcum* var. *monococcum* and *T. ururtu* and tetraploid *T. turgidum* var. *dicoccoides*, respectively. Modern tetraploid durum (pasta) wheat (*T. turgidum* var. *durum*) probably arose from mutations in cultivated emmer.

Hexaploid bread wheat (*Triticum aestivum*) (genomes ABD) has never existed as a wild species and no wild hexaploid wheats are known. It probably arose by hybridization of cultivated emmer with the related wild grass *T. tauschii* (goat grass, also called *Aegilops tauschii* and *Ae. squarossa*). This hybridization probably occurred in south-eastern Turkey about 9000 years ago (Feldman, 1995, Dubcovsky and Dvorak, 2007) and contributed the D genome. All cultivated hexaploid wheats, including spelt, are forms of *T. aestivum*.

A major difference between “ancient” cultivated wheats (einkorn, emmer, spelt) and their wild relatives and modern durum and bread wheats is whether the grain are hulled or free threshing. In hulled wheats the glumes and palea adhere to the grain and the threshed material consists of intact spikelets. By contrast, these structures are removed in “free threshing” durum and bread wheats and the harvested material consists of caryopses. The hulled einkorn, emmer and spelt are together called “faro” in Italy. The reader is referred to Feldman, 1995, Dubcovsky and Dvorak, 2007 and chapters in Elsayed and Wood (2005) for detailed discussions of the evolutionary relationships of wheat species and the characteristics of the “ancient” cultivated forms.

Ancient wheats do not contribute significantly to global wheat production but there is increasing demand, particularly for spelt, due to perceived health benefits. They therefore have high value market value and are frequently produced using traditional or organic farming systems. For example, about 2.5% of the certified organic wheat grain grown in the USA in 2011 was spelt (http://ers.usda.gov/Data-products/organic-production.aspx).

Wild tetraploid and diploid wheats can be readily crossed with their cultivated counterparts to transfer genes and traits, as can cultivated spelt and bread wheats. In addition, genes and traits can be transferred between species with different ploidy levels (Mujeeb-Kazi et al., 2013). It is also possible to “resynthesize” hexaploid wheat, by crossing cultivated durum wheat with diploid *T. tauschii* (Ogbonnaya et al., 2013).

Despite its recent origin, bread wheat shows immense diversity. Feldman (1995) estimated the existence of about 25,000 cultivars/lines and the true number is likely to be substantially greater than this. A survey of accessions in gene banks carried out in 2010 reported a total of over 850,000 accessions of “wheat” (including advanced and old cultivars, land races, breeding lines and wild species) in 230 collections, the largest being over 110,000 accessions held at CIMMYT in Mexico (http://www.fao.org/docrep/013/i1500e/i1500e12.pdf). This represents an immense resource for wheat improvement.

### 3.2 Exploiting genetic diversity in gluten proteins to reduce coeliac toxicity

The improvement of wheat and other crops by plant breeding is based on the identification of genetic variation in traits of interest and incorporating this variation into lines which are commercially competitive in terms of their yield, agronomic performance and quality. In the case of coeliac toxicity, the trait of interest is the number and distribution of coeliac toxic sequences (epitopes). It is therefore logical to determine the relative distribution of coeliac epitopes within different gluten protein types (which is discussed above), and how this distribution varies between proteins coded by the three genomes of hexaploid bread wheat and between the genotypes, including modern commercial cultivars, exotic lines from other parts of the world and “land races” which were cultivated before the use of modern intensive breeding.

#### 3.2.1 α-gliadins

As the most coeliac-active T-cell epitopes are present on the α-gliadins, emphasis has been placed on exploring differences in the amounts and sequences of proteins of this class. Kasarda et al. (1976) used genetic stocks (inter-varietal substitution lines and nullisomic-tetrasomic lines) to map the highly coeliac-active A-gliadin fraction (which comprises α-gliadins) to genes present on chromosome 6A and it was suggested that nullisomic-tetrasomic lines could be used to produce bread for people with coeliac disease (Tucić et al., 1978). Support for this was provided by a preliminary study, in which no adverse reactions were reported in two patients who were fed bread made with nulli-6A wheat (Kasarda et al., 1978). However, Ciclitira et al., 1980a, Ciclitira et al., 1980b subsequently showed adverse reactions when oral challenges were carried out in two coeliac patients fed bread made with either nulli-6A tetra-6D or nulli-6A tetra-6B wheat. Nevertheless, a more recent *in vitro* study using coeliac mucosa and peptic-tryptic digests of total gluten showed a significant reduction in toxicity in in a naturally occurring mutant line which lacked the *Gli-A2* locus on chromosome 6A (Frisoni et al., 1995). It should be noted that this line differed from those used by Kasarda et al. (1978) and Ciclitira et al., 1980a, Ciclitira et al., 1980b in that the absence of the α-type gliadins encoded by the *Gli-A2* locus was not accompanied by compensatory increases in the α-type gliadins encoded by *Gli-B2* or *Gli-D2*. It therefore supported the initial suggestion that the use of lines lacking in α-gliadins encoded by chromosome 6A could contribute to the production of wheat with reduced coeliac toxicity.

Carroccio et al. (2011) have since described the production of a wheat line (C1173) which lacks the α-type gliadins encoded by *Gli-A2* locus and also γ-gliadins and ω-gliadins encode by the complex *Gli-D1/Glu-D3* locus. Prolamins from this line showed significantly lower toxicity *in vitro* than a similar fraction from the control line San Pastore.

van den Broeck et al. (2009) have screened gluten protein fractions from lines of Chinese Spring wheat with partial deletions of the long and short arms of the group 6 chromosomes using monoclonal antibodies that recognise T-cell epitopes. Loss of the α-gliadin locus from the short arm of chromosome 6D resulted in a significant decrease in the presence of T-cell stimulatory epitopes but also a significant loss of dough functionality. This is consistent with the studies of van Herpen et al. (2006) who showed that T-cell stimulatory epitopes were more abundant in α-gliadins encoded by the D genome, and Molberg et al. (2005) who demonstrated that the immunodominant 33mer fragment of α-gliadin was encoded by chromosome 6D (and hence absent from diploid einkorn and tetraploid wheats). A subsequent study also showed that the detrimental effect of the loss of chromosome 1DS on functionality could be compensated for by adding coeliac-safe avenin proteins from oats (van den Broeck et al., 2011).

Variation in the distribution of coeliac toxic motifs between wheat genomes, genotypes and species has been reported in a series of studies, particularly from workers at Plant Research International, Wageningen (NL). Early work from this group in collaboration with the group of Frits Koning at the University of Leiden (Spaenij-Dekking et al., 2005) surveyed the distribution of T-cell stimulatory epitopes in gluten protein sequences in the Uniprot protein sequence database. This showed that T-cell stimulatory sequences were present in 66% (19/29) of α-gliadin sequences.

More detailed studies of the α-gliadin gene family were reported by van Herpen et al. (2006) who combined data on bread wheat from public databases with the cloning and sequencing of genomic DNA from diploid wheats with ancestral A, B and D genomes. The use of genomic DNA meant that both expressed genes and silent pseudogenes were amplified from the diploid wheats, with at least some of the latter being distinguishable by the presence of in frame stop codons. Analysis of the open reading frames of the putative expressed genes (ie those without in frame stop codons) for T-cell stimulatory epitopes showed that two epitopes were present in all 15 α-gliadin sequences from *T. monococcum* and all four epitopes were widely distributed in the 11 α-gliadin sequences from *T. tauschii*. However, some α-gliadin sequences from B genome diploids lacked T-cell stimulatory epitopes. Similar distributions of T-cell stimulatory epitopes were observed when the data from the diploid species were used to assign the bread wheat α-gliadins to genomes.

Mitea et al. (2010) extended this approach, by analysing over 3000 expressed α-gliadin sequences from 11 bread wheat cultivars that were available in public databases. About 40% of the transcripts were assigned to *Gli-A2*, 25% to *Gli-B2* and 35% to *Gli-D2.* The sequences were screened for the presence of five peptide sequences involved in coeliac disease (DQ2-Glia-α1, DQ2-Glia-α2, DQ2-Glia-α3, DQ8-Glia-α1 and P31-43). Variant forms of these epitopes were then tested as synthetic peptides against α-gliadin specific T-cell clones. This demonstrated that several naturally occurring amino acid substitutions eliminated the antigenic activity of the peptides. In particular, naturally occurring and introduced substitutions of serine for proline at specific positions in the α-gliadin epitopes abolished their antigenic properties. The authors therefore suggested that the selection of such mutations constituted a universal approach to eliminate coeliac-toxicity from wheat cultivars.

However, van Herpen et al. (2006) noted that differences in gene expression levels must also be taken into account and screening of gluten protein fractions for T-cell stimulatory epitopes using T-cell based and monoclonal antibody assays showed that differences in levels of expression occurred both within and between species with different genome constitutions (Spaenij-Dekking et al., 2005). Transcriptome analysis also showed wide differences between the expression of genes encoded by chromosomes A, B and D in bread and durum wheats. For example, the expression of genes at the *Gli-A2* locus varied from 12% to 58% of total α-gliadin transcripts in landrace genotypes (Salentijn et al., 2009).

As discussed above, the absence of the D genome from durum wheat could result in lower coeliac activity due to the absence of the T-cell stimulatory epitopes at the *Gli-D2* locus. van den Broeck et al. (2010a) therefore screened 103 accessions of tetraploid wheat by immunoblotting of gluten protein extracts with monoclonal antibodies against the Glia-α9 and Glia-α20 epitopes. This identified three accessions with significantly reduced levels of both epitopes. Further analysis of 61 durum wheat accessions by high throughput transcript sequencing similarly identified some accessions with lower abundances of transcripts containing coeliac disease epitopes (Salentjin et al., 2013).

Finally, van den Broeck et al. (2010b) compared the abundance of the major Glia-A9 coeliac disease epitope in 36 modern wheat cultivars and 50 land races by immunoblotting of gluten protein extracts, using the minor Glia-A20 epitope as a technical reference. The modern cultivars tended to show higher reactivity with the Glia-A9 antibody and lower reaction with the Glia-A20 antibody, although lines showing high and low reactions with both antibodies were present in both sets of germplasm.

#### 3.2.2 Other gluten proteins

Although impressive progress has been made with identifying variation in the abundances of coeliac disease epitopes in α-gliadins, it must be borne in mind that other groups of gluten protein also contain coeliac active sequences.

This was demonstrated in the survey of gluten protein sequences in the Uniprot protein sequence database by Spaenij-Dekking et al. (2005) which is referred to above. They showed that T-cell stimulatory epitopes were present in all γ-gliadin sequences (17/17), in 95.5% (21/22) of HMW subunit sequences and in 5% of LMW subunit sequences (3/57), in addition to 66% (19/29) of α-gliadin sequences. Similarly, the analysis of Chinese Spring deletion lines reported by van der Broeck et al. (2009) showed that the loss of gliadins and LMW subunits encoded by the short arm of chromosome 1DS removed T-cell epitopes but had little impact on functionality (in contrast to the deletion of the α-gliadin genes on chromosome 6D).

Salentijn et al. (2012) assigned γ-gliadin transcript sequences to bread wheat genomes based on comparisons with genomic sequences from related diploid sequences. This showed that γ-gliadins encoded by the *Gli-D1* locus accounted for almost half of the total γ-gliadin transcripts in bread wheat and also had higher contents of coeliac disease epitopes than the γ-gliadins encoded by *Gli-A1* and *Gli-B1*.

### 3.3 Developing coeliac-safe wheat

The detailed studies discussed above clearly demonstrate that there is extensive variation in the occurrence of coeliac-toxic epitopes within and between the sequences of gliadins encoded by the three genomes of bread wheat (and the corresponding genomes of diploid and tetraploid species), and in the levels of expression of these proteins. Although in some cases these include the existence of forms that lack the currently defined coeliac epitopes, it cannot be ruled out that they contain sequences which will stimulate a response in some individuals. Nevertheless it should be possible to exploit this variation to develop wheat cultivars with low levels of these epitopes, if not their absence. However, there are several factors to consider.

Firstly, the studies have focused on a small number of major epitopes in α-gliadins. Although these are the most important in relation to stimulating a response in susceptible individuals, we know that many more epitopes exist (as discussed above) (Sollid et al., 2012).

Secondly, the gliadins and glutenins are encoded by genes in complex multigenic loci and recombination within these loci occurs only rarely. Hence, it will be necessary to select for groups of gluten proteins rather than single components. It will also be necessary to develop tools to enable wheat breeders to select appropriate progeny in their programmes.

Thirdly, the impact of such selection on the yield and processing quality of the lines must be considered, as discussed below.

## 4 Reducing coeliac toxicity by molecular breeding

### 4.1 Mutagenesis

The treatment of plants with radiation or chemical agents to induce mutations is an effective way to generate novel genetic diversity and has been used to produce over 3000 crop cultivars (http://mvgs.iaea.org/Default.aspx). However, whereas morphological traits are readily identified, screening for biochemical or functional traits can be time consuming and expensive. The application to polyploids also poses an additional challenge in that most induced mutations are recessive. Consequently, where gene homologues are expressed on two or more genomes it is necessary to identify and combine mutations in them in order to observe a phenotype.

In recent years, the identification of mutations at the gene sequence level has been facilitated by the development of PCR-based screening, a technology known as TILLING (Targeting Induced Local Lesions in Genomes) (McCallum et al., 2000). Seeds are treated with chemical agents such as sodium azide (NaN_3_) or ethyl methane sulphonate (EMS) to induce point mutations which are randomly distributed over the entire genome. These will include mutations of several types: nonsense mutations which may cause loss of gene function, by truncation or loss of expression of corresponding protein; missense mutations which result in the change of an amino acid in the protein encoded by the mutated gene; and silent mutations which have no effect on the protein sequence or functionality. TILLING can also be used identify genetic variation in natural populations, an approach termed EcoTILLING (Comai et al., 2004). Mutagenized populations and TILLING platforms are now widely available for wheat, including einkorn, durum and bread wheats (Slade et al., 2005, Chen et al., 2012, Rawat et al., 2013, Bovina et al., 2014), and this approach has been successfully applied for identifying mutations affecting starch synthesis and composition (Slade et al., 2012, Uauy et al., 2009, Sestili et al., 2010).

However, the application of TILLING to manipulating gluten protein content and composition is clearly more challenging due to the complexity of the gluten protein loci with multiple expressed genes. It is clearly not realistic to attempt to modify the amino acid sequences of over 50 expressed gluten proteins but it may be possible to specifically up- and/or down-regulate the genes encoding specific groups or families of gluten proteins to manipulate the balance of coeliac toxic to not-toxic proteins. Some success has been achieved by using this approach in transgenics (below).

### 4.2 Transgenesis

The transformation of both bread and durum wheats is now established in a number of laboratories worldwide, using either biolistics or *Agrobacterium*-mediated systems (see, for example, Wu et al., 2009, Sparks and Jones, 2009). This allows both the addition of novel genes, and the down-regulation of endogenous genes using RNAi technology.

This approach was first used by Becker and co-workers (Becker et al., 2006, Becker and Folck, 2006, Becker et al., 2012, Wieser et al., 2006a, Wieser et al., 2006b), who reported down-regulation of α-gliadins in a series of RNAi lines. Wieser et al., 2006a, Wieser et al., 2006b and Becker et al. (2012) reported detailed analyses of flour from a mixture of two transgenic lines, in which α-gliadins were reduced by over 60% compared to the control cultivar. The down-regulation of α-gliadins was associated with compensatory increases in albumins, globulins, other gliadins and LMW subunits, with an overall reduction in total gluten proteins of about 9%. Whereas the dough resistance and extensibility of the two lines were similar, the transgenic line had stronger gluten: this probably related to a decrease in the gliadin:glutenin ratio resulting from the decrease in α-gliadins. The volume of bread baked from the transgenic line was also slightly lower, but the crumb structure and appearance similar. Silencing of the ω5-gliadins has also been achieved by transgenesis, with improvement in dough mixing quality. Such lines would be beneficial for patients with WDEIA and food allergy to wheat (Altenbach and Allen, 2011, Altenbach et al., 2014).

More extensive studies have been reported by the group of Francisco Barro, who generated two series of lines, with down-regulation of only γ-gliadins (Gil-Humanes et al., 2008, Piston et al., 2011) or all gliadins (α-, γ- and ω-) (and in one line also LMW subunits) (Gil-Humanes et al., 2010, Gil-Humanes et al., 2011). This work has been recently reviewed in detail in this journal (Rosell et al., 2014) and will therefore only be briefly described here. The γ-gliadin RNAi lines showed between 65% and 97% reduction in the target proteins, with some effects on the mixing and bread making performance. However, as with the α-gliadin suppressed lines discussed above, they also had greater dough strength and resistance to over-mixing (Gil-Humanes et al., 2012b, Gil-Humanes et al., 2012a, Gil-Humanes et al., 2012a, Gil-Humanes et al., 2012b). The second series of lines had reductions of between 60% and 88% in their contents of all gliadins. The transgenic lines generally had weaker dough than the control, but some also had greater stability (which should confer greater tolerance to over-mixing (Gil-Humanes et al., 2014b). There was also a severe effect on loaf volume, which was reduced by between about 20% and 30% compared to the controls, but the sensory properties were similar to the controls (Gil-Humanes et al., 2014a). However, tests with T-cell clones derived from intestinal lesions of coeliac patients showed that there was also an almost complete suppression of disease-related T-cell epitopes (Gil-Hermanes et al., 2010).

Whereas the studies described above directly targeted the expression of gluten protein genes, Wen et al. (2012) used RNAi to target the gene encoding a demethylase enzyme responsible for the transcriptional deregulation of gliadin and LMW subunit genes. This resulted in between 45% and 76% suppression of the target proteins, with some lines also showing reductions in HMW subunits. The impact of this suppression on celiac toxicity or functionality was not determined.

### 4.3 Genome editing

The term genome editing is applied to a new range of technologies in which highly specific changes are made to genomes without leaving any “footprint” in terms of the presence of foreign DNA. They are based on the use of site-directed nucleases that are engineered to cause breaks at specific sequences in the genome. These breaks are then repaired by the plants' own mechanisms, but these are very prone to errors resulting in a high frequency of mutations. Hence, the variation generated cannot be distinguished from mutations which occur spontaneously at low frequencies or are induced by mutagenesis. Three major classes of nucleases are being exploited: zinc-finger nucleases (ZFNs), transcription activator-like nucleases (TALENS) and clustered regularly interspaced short palindromic repeats (CRISPR) nucleases. The foreign DNA encoding the engineered nucleases may be present on a plasmid or integrated into the genome. In either case the foreign DNA is lost due to segregation leaving only the mutations in the genome. For a more detailed discussion see Jones (2015).

Although transgenesis is required to introduce the nuclease genes into the plants, the absence of foreign DNA from the products, and the fact that the modified sequences cannot be distinguished from other mutations, raises the question of whether the products should be defined as “transgenic” or not (Jones, 2015). This is, in essence, a philosophical question about whether the process or the product should determine the definition.

The application of genome editing to wheat is still in its infancy, but herbicide resistant canola produced by gene editing has already been launched for growth in North America (http://cibus.com/press/press111914.phb).

Although several research groups are currently exploring the application of genome editing to reduce coeliac activity the challenge posed by the presence of multiple genes and expressed proteins means that this is very much a long term target.

## 5 Conclusions: breeding coeliac-safe wheat

Developing coeliac-safe is clearly a realistic prospect, although a combination of technologies may be required (classical breeding and molecular) with a large investment of time and financial support. However, there are major challenges to wide adoption as a crop.

Firstly, it is necessary to retain acceptable processing quality. This is not trivial, as the gluten proteins underpin most systems for wheat processing, including making bread, other baked goods, pasta and noodles. Although work carried out so far indicates that acceptable products may be made using experimental-scale processing, it is a much greater challenge to scale this up to industrial scale production.

Wheat is a critical component of the diet in many low-income countries and, as for other staple commodity crops, availability is highly sensitive to fluctuations in price. The consequences of price rises in such crops are illustrated by the civil unrest observed in a number of countries as a response to increases in food costs (Schneider, 2008). The high cost of cultivar development, increased raw material costs associated with detrimental effects on agronomic performance and yield, and increased processing costs will almost certainly combine to result in a high cost product, at least for the short to medium term. Hence, it likely to be more appropriate for marketing in highly developed economies, as a more attractive alternative to current gluten free products for coeliac patients, rather than wider adoption to reduce the exposure of populations to T-cell stimulatory epitopes and hence the incidence of coeliac disease.

## Figures and Tables

**Table 1:** Summary of the major cultivated and wild species of wheat (based on Feldman, 1995).

Ploidy genome	Wild species	Cultivated species	
diploid			
D	*T. tauschii*		
A	*T. urartu*		
A	*T. monocucum* var. *boeticum*	*T. monococum* var. *monococum*	einkorn
tetraploid			
A B	*T. turgidum* var. *dicoccoides*	*T. turgidum* var. *dicoccum*	emmer
		*T. turgidum* var. *durum*	durum^a^
hexaploid			
A B D		*T. aestivum* var. *aestivum*	bread^a^ wheat
		*T. aestivum* var. *spelta*	spelt

a Free threshing forms.

**Table 2:** List of coeliac disease relevant T-cell epitopes from wheat, barley and rye. Glutamine residues deamidated by tissue transglutaminase are shown in bold, additional glutamine residues targeted by transglutaminase are underlined. The sequences were used to map the epitopes on to prolamin sequences from the GenBank database, which are shown in Fig. 3. Adapted from Sollid et al. (2012).

Epitope	Sequence
DQ2.5 restricted epitopes	
Wheat	
DQ2.5-glia-α1a	PFPQP**Q**LPY
DQ2.5-glia-α1b	PYPQP**Q**LPY
DQ2.5-glia-α2	PQP**Q**LPYPQ
DQ2.5-glia-α3	FRP**Q**QPYPQ
DQ2.5-glia-γ1	PQQSFP**Q**QQ
DQ2.5-glia-γ2	IQP**Q**QPAQL
DQ2.5-glia-γ3	QQP**Q**QPYPQ
DQ2.5-glia-γ4a	SQP**Q**Q**Q**FPQ
DQ2.5-glia-γ4b	PQP**Q**Q**Q**FPQ
DQ2.5-glia-γ4c	QQP**Q**QPFPQ
DQ2.5-glia-γ4d	PQP**Q**QPFCQ
DQ2.5-glia-γ5	QQPFP**Q**QPQ
DQ2.5-glia-ω1	PFPQP**Q**QPF
DQ2.5-glia-ω2	PQP**Q**QPFPW
DQ2.5-glut-L1	PFS**Q**Q**Q**QPV
DQ2.5-glut-L2	FSQQQ**Q**SPF
Barley	
DQ2.5-hor-1	PFPFP**Q**QPF
DQ2.5-hor-2	PQP**Q**QPFPQ
DQ2.5-hor-3	PIP**Q**QPQPY
Rye	
DQ2.5-sec-1	PFPQP**Q**QPF
DQ2.5-sec-2	PQP**Q**QPFPQ
DQ2.2 restricted epitopes	
Wheat	
DQ2.2-glut-L1	PFS**Q**Q**Q**QPV
DQ8 restricted epitopes	
Wheat	
DQ8-glia-α1	**Q**GSFQPSQ**Q**
DQ8-glia-γ1a	**Q**QPQQPFPQ
DQ8-glia-γ1b	**Q**QPQQPYP**Q**
DQ8-glut-H1	QGYYPTSPQ
DQ8.5 restricted epitopes	
Wheat	
DQ8.5-glia-α1	**Q**GSFQPSQ**Q**
DQ8.5-glia-γ1	PQQSFP**Q**Q**Q**
DQ8.5-glut-H1	QGYYPTSPQ
